# Impacts of selected stimulation patterns on the perception threshold in electrocutaneous stimulation

**DOI:** 10.1186/1743-0003-8-9

**Published:** 2011-02-09

**Authors:** Bo Geng, Ken Yoshida, Winnie Jensen

**Affiliations:** 1 Center for Sensory-Motor Interaction, Department of Health Science and Technology, Aalborg University, Fredrik Bajers vej 7 D, Aalborg Øst, Denmark; 2 Biomedical Engineering Department, Indiana University-Purdue University Indianapolis, 723 W. Michigan St, Indianapolis, USA

## Abstract

**Background:**

Consistency is one of the most important concerns to convey stable artificially induced sensory feedback. However, the constancy of perceived sensations cannot be guaranteed, as the artificially evoked sensation is a function of the interaction of stimulation parameters. The hypothesis of this study is that the selected stimulation parameters in multi-electrode cutaneous stimulation have significant impacts on the perception threshold.

**Methods:**

The investigated parameters included the stimulated location, the number of active electrodes, the number of pulses, and the interleaved time between a pair of electrodes. Biphasic, rectangular pulses were applied via five surface electrodes placed on the forearm of 12 healthy subjects.

**Results:**

Our main findings were: 1) the perception thresholds at the five stimulated locations were significantly different (p < 0.0001), 2) dual-channel simultaneous stimulation lowered the perception thresholds and led to smaller variance in perception thresholds compared to single-channel stimulation, 3) the perception threshold was inversely related to the number of pulses, and 4) the perception threshold increased with increasing interleaved time when the interleaved time between two electrodes was below 500 μs.

**Conclusions:**

To maintain a consistent perception threshold, our findings indicate that dual-channel simultaneous stimulation with at least five pulses should be used, and that the interleaved time between two electrodes should be longer than 500 μs. We believe that these findings have implications for design of reliable sensory feedback codes.

## Background

Human beings sense the external environment by exteroceptors and proprioceptors embedded throughout the body, and the receptors are wired to the central nervous system (CNS) via peripheral nerves. Injuries to the nervous system, for example transection of nerves following the amputation of a limb, not only impair motor function but also result in abnormal sensory feedback or neuropathic pain [1]. In those with upper limb amputation, proprioceptive, kinesthetic and tactile feedback from the missing arm/hand is severely degraded. Use of artificial arm/hand prostheses may restore some of the motor function. Of equal importance, restoring some sensory feedback would significantly enhance the user acceptance of prosthetic devices [2-5]. Partly or complete rehabilitation of sensory function of upper limb can significantly improve the quality of life for the affected population.

Sensory feedback can be artificially induced using, e.g., mechanical indentation, intraneural electrical stimulation or electrocutaneous stimulation [6-8]. Among these methods, eletrocutaneous stimulation has been widely used due to its non-invasiveness and capability of producing a sensation whose frequency and intensity can be reliably controlled [9,10]. A number of successful applications of electrocutaneous stimulation in the sensory feedback systems of artificial arm/hand were reported [11-15].

Van Doren et al [2] stated that "a successful sensory feedback system must incorporate a sensory substitute that is the right type and the right magnitude." As such, it is important to maintain a consistent strength of the perceived sensation to gain users' confidence in using the arm/hand prostheses. However, consistent sensory feedback cannot be guaranteed due to many factors, e.g., skin adaptation of sustained stimulation, impedance changes caused by skin reaction. It is a non-linear relationship between stimulation parameters and output sensory responses [9]. This non-linear relation constitutes the main obstacle and challenge today to produce a reliable sensory feedback.

Sensory feedback coding is referred to as the rule by which stimulus parameter modulation is mapped to sensory modulation [16]. The input to this mapping may be modulation of the stimulus amplitude, pulse duration, frequency etc. and the output is the evoked sensation. The strength of the perceived sensation is dependent on the perception threshold, which is varying with different stimulation patterns.

People have investigated the effects of stimulation parameters on the perception threshold in single-channel stimulation. For instance, an inverse relationship was found between the perception threshold and the pulse duration [17]. A study on excitation of sensory nerve indicated that the sensory threshold was higher in the leg than in the forearm [18]. However, to our knowledge, few work can be found in public literature on the perception threshold in dual-channel electrical stimulation. Since dual-channel stimulation has proved to increase the information transfer rate in sensory communication by introducing additional parameters (e.g., the number of active electrodes, the timing between electrodes) into the coding rule set [10], it is important to further study the effect of different dual-channel stimulation patterns on the perception threshold. The perception thresholds on the forearm skin were examined since stimulation of the peripheral nerve close to the missing arm/hand likely produces more intuitive sensory feedback to the amputee patients.

This study investigated the effect of selected stimulation parameters on perception threshold, including the stimulated location, the number of pulses, and the interleaved time between a pair of electrodes. We also evaluated the effect of the number of active electrodes by comparing the perception thresholds in single-channel stimulation with dual-channel stimulation. The hypothesis to be tested in this study is that, the investigated stimulation parameters have significant impacts on perception threshold due to different electrode configurations and different amount of injected charge contributing to either skin physiological variability or ionic micro-environment modification resulted from varied electric field. In addition, the gender difference in perception threshold was also examined.

## Methods

### Subjects

12 healthy human subjects (6 males and 6 females, age 22-39 years, mean 29.1 years) participated in the study. The number of subjects included was determined by the power test performed along the experiments. When the power of statistical comparisons all exceeded 0.8, our recruitment of more subjects stopped. All subjects signed an informed consent prior to the experiment. The experimental protocol was in accordance with the Declaration of Helsinki and approved by the Danish Local Ethics Committee (approval no.: N-20090009). The subjects had no visible skin diseases in the forearm and no known history of neurological or psychological disorders.

### Experimental setup

**Figure 1:**
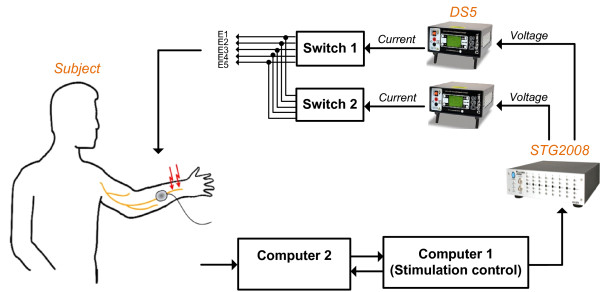
**Experimental setup for the perception threshold measurement**. The experimental procedure was executed on a computerized platform. The stimulation profiles were configured through the stimulation control software residing in *'****Computer 1****'. *The STG2008 generated analog voltage output, and then the DS5s translated the voltage signal into current signal. The stimuli were delivered to one or two electrodes selected by the '***Switches***'. The subject answered whether or not they perceived a stimulus through a Graphical User Interface displayed in '***Computer 2***'.

Figure 1 shows the schematic of the experimental setup. The stimulation patterns were configured through the stimulation control software residing in ***'Computer 1'***. The stimulus generator STG2008 (Multi Channel Systems, Reutlingen, Germany) generated single-channel or dual-channel analog voltage output. The voltage-to-current converters DS5s (Digitimer, Hertfordshire, UK) then translated the voltage signal into isolated current stimuli. The stimuli were delivered to one or two electrodes selected by the two switches. The subject chose '***Yes***' if he/she perceived the stimulation, otherwise chose '***No***' through a Graphical User Interface (GUI) displayed in **'*Computer 2***'. When the subject submitted the answer, ***'Computer 1' ***received an acknowledgement signal and the next stimulation was delivered after 5 seconds. During the experiment, the stimulus information was blind to the subjects.

**Figure 2:**
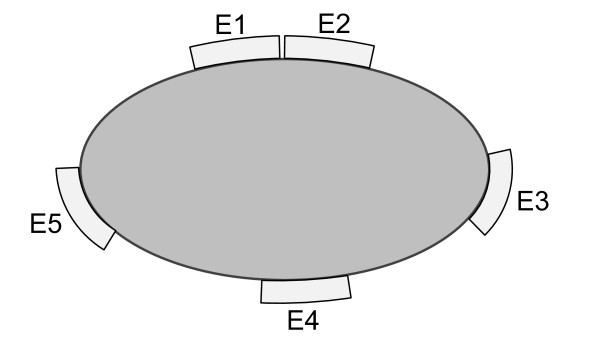
**Schematic of the cross-section view of the electrode placement**. Five electrodes were placed around the forearm (view from the distal side). The right side (E3) corresponds to the radial side. The upper side corresponds to the ventral side (E1 and E2).

Five Ambu Neuroline 700 solid gel electrodes (skin contact size: 20 mm × 15 mm) were placed around the left forearm 5 cm distant to the antecubital crease (Figure 2). For brevity, the five electrodes (or channels) are referred to as: E1, E2, E3, E4 and E5. The location of the five electrodes was standardized among subjects according to the following rules: 1) E1 was placed over the median nerve, 2) E2 was placed laterally adjacent to E1, 3) E3, E4, and E5 were equally spaced between E2 and E1. The common reference electrode was positioned over the ulnar styloid process in the left forearm. The median nerve was identified by applying electrical stimulation with moderate intensity. The place where the evoked sensation projected to the thenar eminence, the thumb and/or the index and/or the middle finger was then identified as the location of the median nerve. E1 and E2 were placed adjacently with the purpose to examine the influence of the distance between electrodes. The skin area identified for stimulation was prepared with a water soaked cotton cloth to decrease the impedance and thereby facilitate stimulus current conduction.

A symmetric, biphasic, rectangular waveform was applied since it had the lowest total charge among five commonly used waveforms [18]. The pulse durations of 100 μs, 200 μs and 500 μs were first tested in a pilot experiment. Pulse duration of 200 μs was finally chosen as it produced the least 'prickly' sensation. The frequency of 20 Hz was used, since it was found that 20 Hz might be the optimal frequency for sensory communication as the maximum frequency discrimination occurred near 20 Hz [19].

### Stimulation application

**Figure 3:**
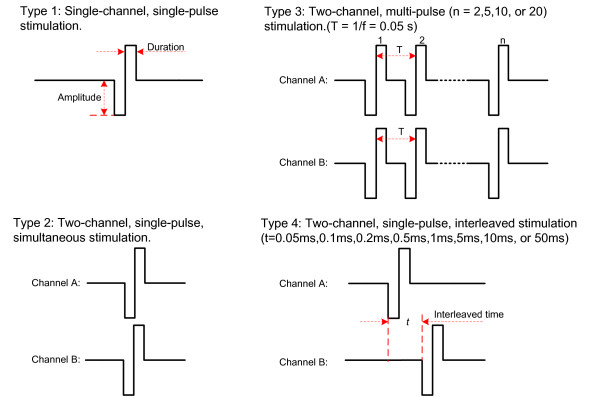
**Illustration of the four types of stimulation patterns**. Left-Top (Type 1): Single-channel, single-pulse stimulation. Left-bottom (Type 2): Dual-channel, single-pulse simultaneous stimulation. Right-Top (Type 3): Dual-channel, multi-pulse, simultaneous stimulation with *n *pulses. Right-Bottom (Type 4): Dual-channel, single-pulse stimulation with interleaved time *t*.

Four types of stimulation patterns were applied (Figure 3). In Type 1, a single-pulse stimulus was applied to individual electrodes. In Type 2, two single-pulse stimuli were applied to a pair of electrodes simultaneously. Seven out of ten possible electrode pair combinations were measured, i.e., E1&E2, E1&E3, E1&E4, E1&E5, E3&E4, E3&E5 and E4&E5. The remaining three, i.e., E2&E3, E2&E4 and E2&E5, were ignored, as we found that the combinations with E2 resulted in similar perception thresholds to those combinations with E1 (results not shown). In Type 3, two multi-pulse stimuli (*n *= 2, 5, 10, or 20) were applied to a pair of electrodes simultaneously. Considering the amount of time needed to measure all combinations, only three pairs were measured in the Type 3 stimulation, i.e., E1&E2, E1&E4, and E3&E5. These three were selected because they include all the five electrode locations and varying inter-electrode distances. In the Type 4, two single-pulse stimuli were applied to two electrodes with an interleaved time (*t *= 0.05 ms, 0.1 ms, 0.2 ms, 0.5 ms, 1 ms, 5 ms, 10 ms, or 50 ms). The same three electrode pair combinations were measured for the Type 4 stimulation. As such, the perception thresholds of totally 48 different stimulation parameter combinations were measured for each subject.

### Perception threshold measurement

**Figure 4:**
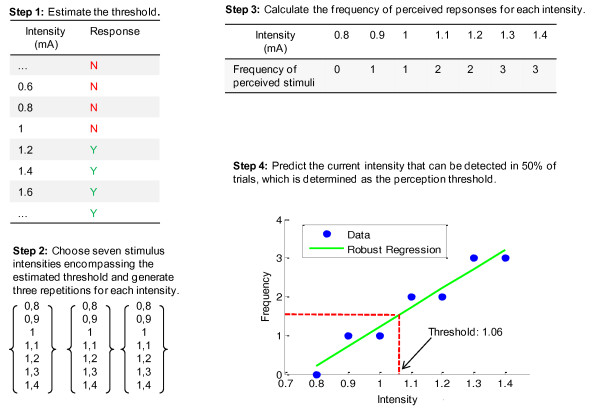
**Measurement of the perception threshold with an example data from a subject**. Step 1: The perception threshold was roughly estimated to be 1.1 mA, since 1.0 mA was the intensity last perceived and 1.2 mA was the one first perceived. Step 2: Seven intensities were chosen in the range of 0.8~1.4 mA. Three repetitions produced 21 stimuli. Step 3: The frequency of 'perceived' responses were calculated for each of the seven intensities. Step 4: The perception threshold was determined to be 1.06 mA by predicting the intensity that would be detected in 50% of trials (i.e., 1.5 times).

The perception threshold was defined as the current amplitude that a subject could just barely detect. The amplitude was measured on the activation side (i.e., negative phase) of the biphasic pulse. The measurement of the perception threshold proceeded as illustrated in Figure 4. First, the perception threshold was roughly estimated by applying a series of ascending-amplitude stimuli with a step size 0.2 mA. The average of the amplitude of the last 'not-perceived' and the first 'perceived' stimulus was then identified as the approximate threshold. Then, seven stimuli were chosen, with amplitudes in a range encompassing the approximate threshold just identified, but with a smaller step size of 0.1 mA. Afterwards, three repetitions of the seven amplitudes was mixed and presented to the subject in a pseudo-random order (i.e., totally 21 stimuli). After each stimulus presentation, the subject reported whether or not the stimulus was perceived. Finally, the frequencies of 'perceived' and 'not-perceived' reports were calculated for each of the seven intensities. Due to the variability in the biological sensor systems and psychological fluctuation, the plot of frequency against intensity is typically not all-or-none curve [20]. In general, a lower amplitude was occasionally perceived and a higher amplitude was more often perceived. It was assumed that within the vicinity of the perception threshold, the frequency of 'perceived' responses and the stimulus intensity are linearly correlated [21]. Thus, the perception threshold was determined by predicting the intensity that would be detected in 50 percent of the trials (i.e., 1.5 times). Robust linear regression was used to implement the prediction.

### Data analysis

The effects of the investigated stimulation parameters on the perception threshold were statistically evaluated. Based on the observation of the Q-Q plots of the four individual stimulation types, the perception threshold data were assumed to follow a normal distribution. The independent variables were 'stimulated location', 'channel combination', 'the number of pulses' and 'interleaved time'. The dependent variable was 'perception threshold' in all comparisons. A one-way, repeated analysis of variance (ANOVA) was performed and the F-test was used to test if there was a significant effect of an independent variable. The significance level was chosen to be 0.05. When a significant difference was found, individual pairs of conditions were further compared using multiple pairwise comparisons with Bonferroni adjustment. The relationship between the perception threshold and the number of pulses or the interleaved time was further evaluated by curve fitting.

## Results

### Effect of the stimulated location on the perception threshold

**Figure 5:**
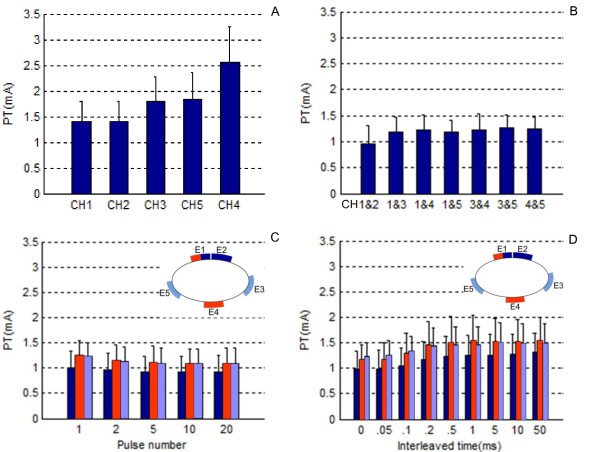
**Mean and standard deviations of the PTs measured from all subjects**. A. PTs in the single-channel, single-pulse stimulation (Type 1); B. PTs in the dual-channel, single-pulse simultaneous stimulation (Type 2); C. PTs in the dual-channel, multi-pulse, simultaneous stimulation (Type 3); D. PTs in the dual-channel, single-pulse, interleaved stimulation (Type 4). Note that the three electrode combinations in C and D are indicated by three different colors in the inset schematic of electrode placement.

The lowest perception threshold (PT) was found at E1 and E2, whereas the highest PT was found at E4 (dorsal side) (see figure 5A). The PTs at E3 and E5 were at the middle level. As such, it appears that the closer the electrode was placed to the median nerve, the lower the PT. The ANOVA result indicated that the stimulated location had a significant effect on the PT (*F *(4, 55) = 12.03, *p <*0.0001, test power = 0.91). Pairwise comparisons were then performed. Table 1 lists the results of the multiple comparisons. The PT at E4 had a significant difference from all other four electrodes.

**Table 1:** Pairwise comparisons of the mean PTs among five electrode sites.

Electrode pair	Significant difference?	P-value	95% CI of mean difference (mA)
(E1, E2)	No	1.000	[-0.19, 0.21]
(E1, E3)	Yes	0.028	[-0.74, -0.03]
(E1, E4)	Yes	0.002	[-1.88, -0.42]
(E1, E5)	No	0.267	[-1.00, 0.16]
(E2, E3)	Yes	0.038	[-0.78, -0.02]
(E2, E4)	Yes	0.001	[-1.84, -0.49]
(E2, E5)	No	0.128	[-0.95, 0.08]
(E3, E4)	Yes	0.017	[-1.41, -0.12]
(E3, E5)	No	1.000	[-0.62, 0.54]
(E4, E5)	Yes	0.026	[0.07, 1.38]

### Effect of the number of active electrodes: single-channel vs. dual-channel stimulation

The PTs in dual-channel stimulation were observed less varying than in single-channel stimulation (see figure 5B). The effect of the number of active electrodes was evaluated by comparing the PT in dual-channel stimulation with the PT in stimulation of either of the two channels. We found that, for pairs of electrodes showing no significant difference in Table 1 (i.e., E1 and E2, E1 and E5, E3 and E5), simultaneous stimulation of the two electrodes reduced the PT. This result verifies that incorporating additional electrodes increased the bandwidth of sensory information transfer.

To examine the effect of the distance between electrodes in dual-channel stimulation, we compared the PT at E1 with the PTs at E1 combined with the other four electrodes individually. Moreover, the PT at E3 was compared with the PT at E3 combined with E4 and E5, respectively. The results of the statistical comparisons are listed in Table 2.

**Table 2:** PT comparisons of single-channel and dual-channel stimulation.

Comparison	Significant difference?	P-value	95% CI of mean difference (mA)	Test Power
E1 vs. E1&E2	Yes	7E-7	[0.37, 0.59]	1.00
E1 vs. E1&E3	Yes	0.012	[0.01, 0.43]	0.80
E1 vs. E1&E4	Yes	0.013	[0.05, 0.36]	0.77
E1 vs. E1&E5	Yes	0.011	[0.08, 0.45]	0.82
E3 vs. E3&E4	Yes	0.004	[0.21, 0.78]	0.94
E3 vs. E3&E5	Yes	0.008	[0.16, 0.77]	0.86

It can be seen that, compared to single-channel stimulation at E1, incorporation of a second channel significantly reduced the PT, irrespective of the distance to E1. The extent of the decrease depended on the distance. The closer the second electrode was placed to E1, the more the PT was reduced. The extent of the PT reduction can be reflected in the 95% confidence interval of the mean difference. A larger mean difference suggests a larger PT decrease. The mean difference of the PT at E1 and at E1&E2 was the highest, since E2 was placed closest to E1. Similarly, the PT mean difference between E3 and E3&E4 was larger than between E3 and E3&E5 since E3 was further away from E5 than E4.

### Effect of the pulse number on the perception threshold

Figure 5C shows the PTs measured in the dual-channel, simultaneous stimulation with five varied pulse numbers. In the bar plot, a slight decline of the PT with the increasing pulse number can be observed. The ANOVA analysis indicates that there was a significant effect of the pulse number on the PT (E1&E2: *F *(4, 55) = 5.96, *p *< 0.01, *test power *= 0.87; E1&E4: *F *(4, 55) = 18.98, *p *< 0.0001, *test power *= 1.00; E3&E5: *F *(4, 55) = 24.26, *p *< 0.0001, *test power *= 1.00).

**Figure 6:**
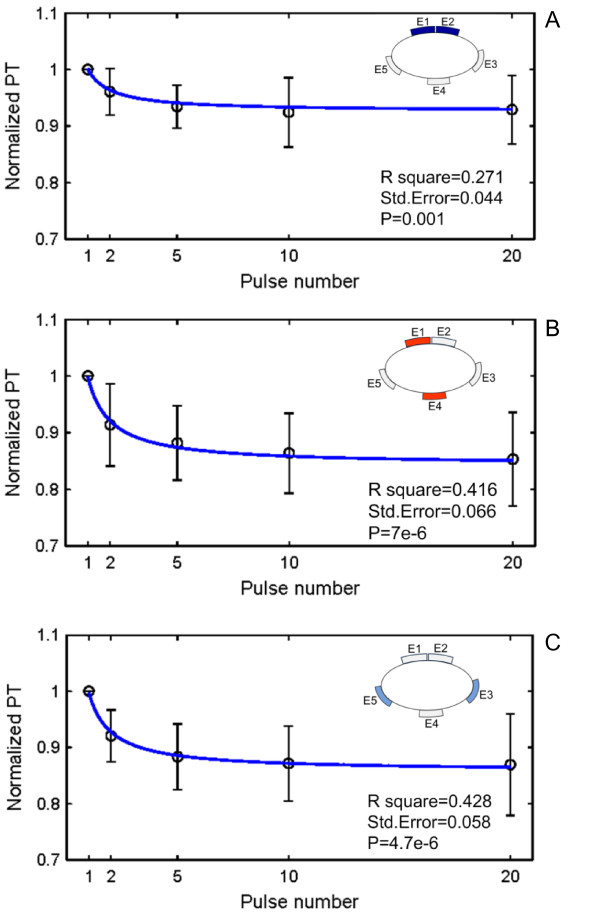
**Relationship between the normalized PT and the number of pulses**. A. E1&E2. B. E1&E4. C. E3&E5. An inverse relationship between the PT and the pulse number *n*: *PT *= *a + b/n *was found in all the three electrode combinations. The highlighted electrodes in the inset schematic indicate the location of stimulated electrodes corresponding to the figure.

Curve fitting was further used to examine the effect of pulse number on the PT (Figure 6). An inverse relationship between the PT and the pulse number *n*: *PT *= *a + b/n *was found in all the three electrode combinations. The results indicate a significant effect of pulse number on the PT. Note that, the data of each subject was normalized to the PT measured with this subject when the pulse number was one, in order to eliminate the effects from other factors.

### Effect of the interleaved time between a pair of electrodes

**Figure 7:**
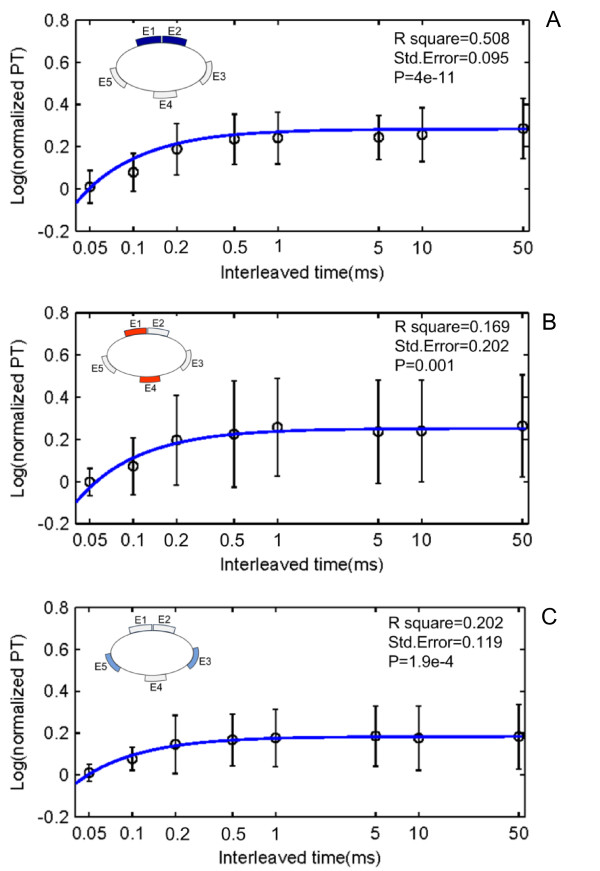
**Relationship between the normalized PTs and the interleaved time between two channels**. A. E1&E2. B. E1&E4. C. E3&E5. A sigmoid relationship was estimated between the PT and the interleaved time *t*: log (*PT*) = *c *+ *d*/*t*. The highlighted electrodes in the inset schematic indicate the location of active electrodes corresponding to the figure.

An increase of the PT with increasing interleaved time between a pair of electrodes was observed in all the three electrode pair combinations (see figure 5D). Curve fitting was used to estimate the relationship between the PT and the interleaved time (see Figure 7). Likewise, the data collected from each subject were normalized by the PT measured with this subject when the interleaved time was 0. A sigmoid relationship was found between the PT and the interleaved time *t*: log (*PT*) = *c *+ *d*/*t*. The results indicate a significant effect of the interleaved time on the PT.

The PT increased with increasing interleaved time and reached a plateau approximately at the point of 500 μs. The ANOVA analysis indicated a significant difference among the PTs below 500 μs (*p *< 0.01, *p *< 0.05, and *p *< 0.05 in E1&E2, E1&E4 and E3&E5, respectively), while no significant difference was found above 500 μs.

### Gender difference

**Figure 8:**
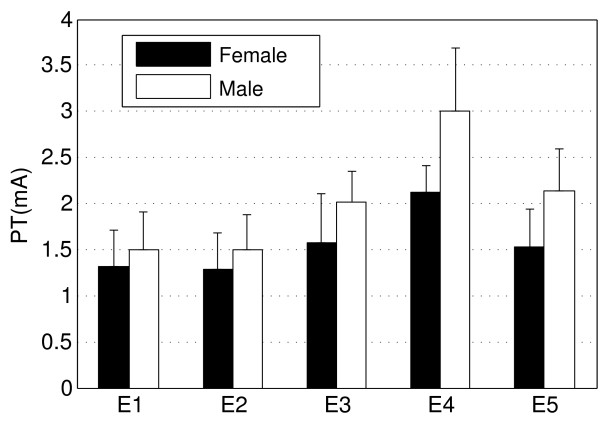
**Bar plot of the perception thresholds in the female and male subjects**. The mean differences between the male and female subjects are 0.18 mA, 0.22 mA, 0.44 mA, 0.88 mA, and 0.61 mA, respectively (E1 to E5).

We also investigated the effect of gender on the PT. Figure 8 shows the mean and standard deviations of the PT in single-channel, single-pulse stimulation. The interesting finding was that, at all five locations, the female subjects exhibited lower PTs than the male subjects. The mean differences between the male and female subjects are 0.18 mA, 0.22 mA, 0.44 mA, 0.88 mA, and 0.61 mA, respectively (from E1 to E5).

## Discussions

Perception of an external stimulus essentially results from the activation of the afferent units present in the skin. The modalities of evoked sensations are determined by the types of activated sensory receptors. In this study, the subjects reported the sensations of touch, light pressure and tingling. Thus, we believe that the activated receptors were likely the hair follicles, Ruffini, or free nerve endings, and the corresponding nerve fibers activated were the Aβ or Aδ [22]. When the dual-channel stimulation was applied to E1 and E2, the subjects could not discriminate the two stimulation sites, which could be explained by the fact that E1 and E2 were located within one receptive field. In the case of the dual-channel stimulation of other electrode pairs, most subjects reported to perceive the stimulation at the channel with lower PT.

The significantly different PTs measured at the five electrode locations suggest that the stimulus amplitude adequate to elicit a sensation varies across the skin. We speculate that the skin impedance may be one of the sources accounting for the PT variations (the skin impedance was not measured in the current study). Human skin tissue can be modeled by an equivalent circuit using multiple resistors and capacitors (see e.g., [23-25]). Skin thickness has an influence on the distance between the stimulus source and the nerve endings in the skin. A larger distance between the current source and the activated nerve endings indicates a higher coupling impedance, which correspondingly results in a higher propagation loss. A higher current amplitude is thus needed to activate the afferent receptor or fibers, and consequently it results in a higher PT. A previous study based on ultrasonic imaging techniques demonstrated that the skin is thicker on the dorsal than volar forearm [26]. Even within the skin area under a surface gel-type electrode, the electrical current density is distributed unevenly due to uneven skin resistivity [27]. The result that E1 and E2 (ventral side) had lower PTs while E4 (dorsal side) had a higher PT, assists to strengthen our speculation that lower skin impedance led to lower PT. Another possible source of the PT variability might be the variations in the density of nerve endings. It can be partly supported by previous studies on tactile afferent units distributed in the forearm, in which the receptive field was found to be varied widely in size [28,29].

No significant difference in the PT was found between E1 and E2, likely because E1 and E2 are closely located and the afferent fibers innervating the skins under E1 and E2 overlap spatially, causing the same set of sensory fibers were activated.

The reduced PTs by simultaneous stimulation at E1&E2 may be explained by spatial summation of the electric fields. More electric charges were injected in dual-channel stimulation than single-channel stimulation, resulting in lower current amplitude required to activate the nerve endings. PT reduction was found also in dual-channel simultaneous stimulation when the two electrodes were located not so close (from 10 cm to 20 cm depending on the forearm size), i.e., E1&E4 or E3&E5. This might be caused by the charge summation occurring centrally, even if distinct sets of nerve fibers were activated peripherally. Another possible explanation is that the receptive field sizes for some types of afferents are fairly large. Although E4 was located farthest away from E1, the groups of cutaneous nerve fibers under E1 and E4 electrodes may still overlap. This speculation may be supported by the fact that the receptive fields of myelinated afferents in the forearm could be up to 210 mm^2 ^[29].

In the dual-channel interleaved stimulation, the interleaved time shorter than 200 μs (i.e., pulse duration) could lead to a temporal overlap of two pulses and consequently a temporal summation of electric fields. The summation caused a lower current for the threshold activation of nerve fibers. When the interleaved time increased above the pulse duration (i.e., 200 μs), no more temporal overlap occurred. However, the transition point did not occur at 200 μs. We consider that, immediately after the pulse overlap period there likely was a 'RC recovery time interval', during which the membrane still contained the charge of the first stimulus, causing that the second stimulus raised the membrane potential above excitation threshold [30].

The PT barely changed when the interleaved time is longer than 500 μs. This imply that the PT may be more stable when the time separation between two electrodes longer than 500 μs. Similarly, a saturation effect was observed when the pulse number was larger than five. This may suggest that a stimulus with at least five pulses is capable of producing more consistent strength of perceived sensation.

This study mainly focused on the effects of different stimulation patterns on the PT within subjects. However, the variation between subjects should not be ignored. One source of the variation between subjects might be resulted from the variability of the body fat percentage from subject to subject (the body fat percentage was not measured in the current study). The body fat percentage is closely associated with the tissue volume conductor, which directly impacts the nerve fiber recruitment. It is well established that women generally have a higher percentage of body fat than men. Since the PTs in the female subjects were shown to be lower than the male subjects, it may imply that the PT was interrelated to the body fat percentage. It should be noted that the conclusion that females have lower PT than males is speculative due to the small sample size. Yet the observation is in accordance with the finding in [31].

The method of constant stimuli was used to measure the PT. In the classical method of constant stimuli, a set of stimulus intensities (usually from 5 to 9) encompassing the actual threshold are chosen and then presented multiple times (usually not less than 20 times) in a pseudo-random order, with each occurring equally frequent. Once the percentage of 'perceived' and 'not perceived' responses to each intensity calculated and plotted against stimulus intensity, the PT is determined by linear interpolation of the stimulus intensity perceived in 50% of present times. The method of constant stimuli is generally considered to provide the most reliable estimate of the PT (see e.g. [21]), as a random presentation of stimuli can efficiently eliminate the possible bias from the subject's anticipation. However, its main drawback is that many times of presentations of each value and tracking of the subject's response is considerably time-consuming, which easily distract the subject's attention. To limit the time consumption and meanwhile maintain measurement accuracy, we reduced the number of presentations of each intensity and compensated the possible accuracy loss by introducing a 'roughly-estimate' procedure. That is, a series of intensities with a bigger step size was used to roughly estimate the threshold, and then around the threshold just estimated, a set of intensities with a smaller step size was presented to the subject multiple times. As such, the procedure optimized the intensity set by adapting the stimuli according to the subject's responses.

Choosing the step size of the stimulus amplitudes is critical since only amplitudes near the threshold can provide useful information. Too big step sizes may overestimate the threshold range in that some of the amplitudes will be too far away from the actual threshold, causing inefficiency. Too small step sizes possibly underestimate the threshold range, leading to biased measurement of the threshold. The optimal amplitude set should be just across the region of sensory fluctuation. In our pilot experiment, five different steps sizes (0.02 mA, 0.05 mA, 0.1 mA, 0.15 mA, 0.2 mA) were tested in single-channel, single-pulse stimulation with one subject. With each step size, the PT was measured several times following the procedure described in the section of perception threshold measurement. It was observed that, in the case of 0.15 mA and 0.2 mA most stimulus intensities were either 'perceived' or 'not perceived' in all three repetitions, which provided limited information for the PT estimation. In the case of 0.02 mA and 0.05 mA, inconsistent PTs were obtained in the measurements. Therefore, 0.1 mA was chosen to be the step size.

This study revealed the influence of the investigated electrical stimulation parameters on the PT on the forearm skin. The results provide insight into the use of electrocutaneous stimulation to induce magnitude-stable sensory feedback in advanced upper limb or hand prostheses. Also, the results give implication for selection of appropriate stimulation parameters for sensory discrimination training program, which can be used to reduce PLP or other chronic limb pain [32,33].

## Conclusions

Within the study on a limited set of stimulation parameters in single-channel and dual-channel stimulation, we conclude that incorporation of a second stimulating electrode reduced the perception threshold. In dual-channel simultaneous stimulation, there is an inverse relationship between the perception threshold and the number of pulses. And the perception threshold is positively related to the time separation in the interleaved stimulation when the interleaved time was shorter than 500 μs. Based on the findings, we propose that dual-channel stimulation with pulse number larger than five, as well as the time separation between two stimuli longer than 500 μs in interleaved stimulation can be used to achieve reliable perception threshold. We also suggest applying the stimulation on the ventral side of the forearm to induce sensory feedback because it has significantly lower PT than the dorsal side. The findings may help develop reliable sensory feedback codes and provide an insight into understanding the neurophysiological substrate of electrocutaneous stimulation.

## Competing interests

The authors declare that they have no competing interests.

## Authors' contributions

BG, WJ and KY designed the experimental protocol. BG performed the experiment, conducted data collection and analysis. KY, WJ and BG outlined the fundamental concepts of the scientific research, and contributed to the preparation of the manuscript. All authors read and approved the final manuscript.
